# Development and evaluation of explainable machine learning models for predicting prognosis in patients with primary biliary cholangitis

**DOI:** 10.3389/fmolb.2026.1900864

**Published:** 2026-07-28

**Authors:** Yifeng Dou, Jiantao Liu

**Affiliations:** 1 Network Information Center, Tianjin Medical University Baodi Hospital, Tianjin, China; 2 Department of Traditional Chinese Medicine, Tianjin Medical University Baodi Hospital, Tianjin, China

**Keywords:** extreme random trees, interpretability, machine learning, prediction, primary biliary cholangitis

## Abstract

**Background:**

This study aimed to develop, validate and evaluate interpretable machine learning models using clinical and laboratory data for prognosis prediction in patients with primary biliary cholangitis (PBC).

**Methods:**

This study included 7905 patients treated for PBC. The cohort data were randomly divided into training and testing sets, with external validation using an additional 2372 patients. Six machine learning models are compared in this research (logistic regression (LR), random forest (RF),Extreme Random Trees (ET), Extreme Gradient Boosting (XGBoost), Lightweight Gradient Boosting Machine (LightGBM), and Multi-Layer Perceptron (MLP)). Feature importance and model interpretation were analyzed using the SHapley Additive exPlanations (SHAP) method.

**Results:**

Ensemble tree models demonstrated significantly superior performance compared to traditional linear models and shallow neural networks in predicting PBC patient prognosis. Among these, the Extreme Random Tree model exhibited optimal predictive efficacy on both the training set (AUC = 0.9898) and external validation set (AUC = 0.9681), while the Random Forest model showed comparable performance with greater stability. At the patient level, SHAP’s force maps and decision trees provided clinically meaningful explanations for the et algorithm. The bilirubin_albumin_ratio emerged as the core feature for predicting PBC prognosis, with bilirubin, n_days, and prothrombin serving as key influencing factors. The influence patterns of these features align closely with clinical and pathological mechanisms.

**Conclusion:**

The ET model constructed in this study enables precise prognosis prediction for PBC patients. After SHAP analysis, it demonstrates good interpretability. The key prognostic features identified by the model provide quantitative evidence for clinically assessing disease severity in PBC patients and offer data support for developing individualized clinical intervention plans.

## 1 Introduction

Primary biliary cholangitis (PBC) is a chronic autoimmune liver disease characterized by progressive, immune-mediated damage to intrahepatic small bile ducts. PBC has an insidious clinical course and highly heterogeneous disease progression. Advanced PBC frequently leads to liver fibrosis, cirrhosis and even liver failure, which imposes a substantial burden on global public health (European Association for the Study of the Liver, 2017; Gao et al., 2020). Global epidemiological data indicate a persistent upward trend in PBC incidence. Prevalence rates in European and American populations have reached 19.1–40.2 per 100,000 individuals. Although slightly lower in Asian populations, the annual growth rate exceeds 5%. Women face a 9–10 times higher risk than men, with peak incidence occurring between ages 40 and 60 (Corpechot et al., 2008; Lleo et al., 2020). Although ursodeoxycholic acid (UDCA) as first-line therapy effectively controls disease progression in 60%–70% of patients, with 10-year survival rates approaching those of healthy individuals, 20%–40% of patients exhibit poor response to UDCA. These patients face increased risks of progression to cirrhosis and hepatocellular carcinoma, with markedly reduced survival rates. with a 10-year mortality rate exceeding 90% (Jorgense et al., 2002; You et al., 2022). Traditional prognostic assessment systems (e.g., ALBI score (Chen et al., 2023), GLOBE score (Sohal and Kowdley, 2023), UK-PBC score (Wan et al., 2025)) primarily rely on linear combinations of limited clinical indicators. They struggle to capture the complex nonlinear relationships and multifactorial interactions involved in PBC disease progression, exhibiting significant limitations in individualized risk stratification and treatment decision-making guidance. There is an urgent need to develop more precise and clinically practical prognostic prediction tools.

Machine learning (ML), with its robust capabilities in feature extraction, nonlinear fitting, and complex pattern recognition, has achieved a leap from basic research to clinical translation in hepatology, offering an innovative technical pathway to address the challenge of PBC prognosis prediction (Gerussi et al., 2025). In recent years, scholars worldwide have conducted systematic explorations on ML applications in PBC. Regarding treatment response prediction, research primarily focuses on first-line ursodeoxycholic acid (UDCA) and second-line obeticholic acid (OCA), with both yielding efficient, validated predictive models through core clinical indicator selection. Zhu et al. developed a model using three independent risk factors—total cholesterol, alkaline phosphatase (ALP), and neutrophil-lymphocyte ratio (NLR)—achieving AUC values of 0.862 and 0.916 in internal and external validation, respectively, enabling precise identification of UDCA non-responders at baseline (Zhu et al., 2024). Kimura et al.'s Extreme Gradient Boosting Tree model, utilizing total bilirubin, total protein, and alanine aminotransferase as core variables, achieved an AUC of 0.856 for predicting UDCA response in a multicenter cohort, significantly outperforming traditional statistical methods (Kimura et al., 2024). Beyond clinical indicators, non-invasive imaging features have also been successfully integrated into UDCA response prediction systems. Zhang et al. constructed a model based on three pre-treatment non-contrast MRI features—hepatic enlargement, high signal intensity around the portal vein on T2-weighted images, and bile duct stricture—achieving 85.4% sensitivity and 61.5% specificity in accurately predicting poor 12-month UDCA biochemical response, offering a new pathway for non-invasive diagnosis and treatment of PBC (Zhang et al., 2024). For OCA, a second-line drug for patients with poor UDCA response,De Vincentis et al. developed the OCA Response Score (ORS) and ORS + model. The ORS incorporates pre-treatment clinical indicators (age, pruritus, cirrhosis, ALP/ULN, etc.), while the ORS + model includes dynamic indicators at 6 months post-treatment. These models achieved predictive performance with C-statistics ranging from 0.70 to 0.88 under different response criteria, providing quantitative tools for stratified second-line treatment in PBC and aiding in the development of personalized clinical treatment plans (Vincentis et al., 2024). Additionally, early studies identified independent risk factors for UDCA non-response, including total bilirubin, albumin, and immunoglobulin M, with the constructed predictive index achieving an AUC of 0.886, laying the foundation for subsequent model optimization and refinement (Shu et al., 2021). In PBC prognosis risk stratification and complication early warning, machine learning has achieved a leap from traditional single-indicator assessment to multidimensional comprehensive modeling, significantly enhancing the accuracy of risk stratification and the foresight of complication early warning. For PBC-related cirrhosis patients, Fu et al. developed a prognostic model using a random forest survival model with cholinesterase, bile acids, white blood cell count, total bilirubin, and albumin as core variables. Internal validation yielded a C-index of 0.7805, enabling precise classification into high- and low-risk groups. The model achieved AUC values of 0.9595, 0.8898, and 0.9088 for predicting 1-, 3-, and 5-year survival, respectively, providing precise quantitative support for targeted treatment strategies, 0.8898, and 0.9088, respectively, providing precise quantitative evidence for targeted treatment strategies (Fu et al., 2024). In cirrhosis complication prediction, Zhou et al. pioneered the integration of psychosocial indicators into PBC risk prediction systems. Using Lasso-logistic regression and decision tree models, they identified the Hamilton Depression Rating Scale 17 (HAMD-17) score as an independent risk factor for cirrhosis development in PBC patients (OR = 1.087). Furthermore, depressive symptom severity was found to correlate significantly with disease severity. This study expanded the scope of indicators incorporated into machine learning models, offering a new perspective for multidimensional risk assessment in PBC (Zhou et al., 2024).

Despite incremental progress in ML applications for PBC research, existing studies exhibit several limitations. These include a lack of model interpretability, with most investigations overly focused on optimizing predictive performance while neglecting the clinical trust crisis stemming from ML models’ “black box” nature. There remains insufficient systematic analysis of feature contribution mechanisms and decision-making logic. Feature engineering (Shil et al., 2026; Bilal et al., 2025) often lacks scientific rigor, with some studies directly modeling raw clinical indicators without adequately considering the skewed distribution characteristics of medical metrics or the clinical correlations between features. The absence of feature derivation and selection strategies grounded in pathological mechanisms limits both predictive efficacy and clinical utility. Performance evaluation systems remain incomplete. Most studies rely solely on basic metrics like AUC and accuracy, failing to incorporate key indicators reflecting clinical net benefit—such as decision curves analysis (DCA) (Montero, 2026) and calibration curves (CC) (Calster et al., 2019), thereby hindering comprehensive assessment of a model’s clinical translation potential.

**FIGURE 1:**
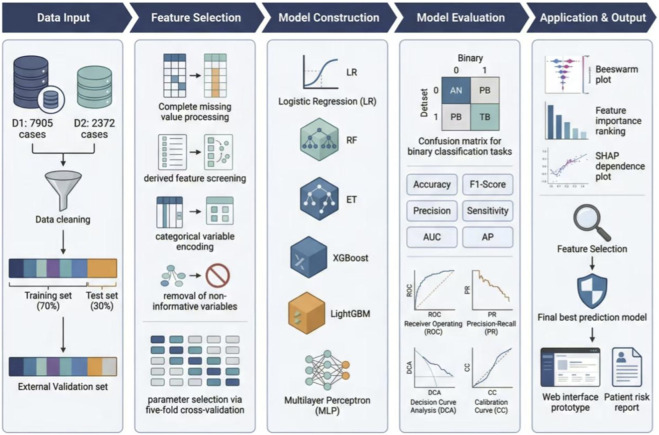
Technical flowchart of the study. The total raw dataset is first split into two fully independent non-overlapping cohorts (Internal cohort D1:7905 cases; External validation cohort D2:2372 cases) with zero patient overlap; only D1 enters data cleaning, feature engineering, train/test split (70% training/30% test), five-fold cross-validation and model construction; D2 is fully held-out for independent external validation without any participation in model training or parameter optimization.

This study features rigorous experimental design with large-sample multi-dataset validation, using 7905 PBC patients for model training and testing and another 2372 independent patients for external cross-dataset validation; it delivers models with better generalization performance and higher clinical reliability than prior small-sample studies, and the comprehensive comparison of six mainstream machine learning algorithms also addresses the lack of horizontal performance evaluation of multiple models in PBC prognostic prediction. A standardized and clinically oriented feature engineering framework is established in accordance with the pathological features of PBC, where differentiated preprocessing methods are applied to categorical variables, skewed continuous indicators and clinically correlated indicators. The newly constructed bilirubin-albumin ratio and comprehensive symptom score can better reflect liver function and disease severity, and recursive feature elimination based on random forests guarantees the clinical interpretability of variables, avoiding the drawback of arbitrary feature selection in previous liver disease-related machine learning research. Differing from most existing “black-box” PBC prediction models that merely pursue prediction accuracy, this work adopts the SHapley Additive exPlanations (SHAP) (Lundberg et al., 2020) algorithm to conduct global feature importance analysis, local decision interpretation and feature dependency analysis, and quantifies the critical thresholds of core prognostic indicators, converting data-driven models into interpretable tools conforming to clinical and pathological principles and improving the clinical credibility of machine learning models. Beyond conventional metrics such as AUC and accuracy, this research further employs decision curve analysis and calibration curves to assess clinical net benefit and prediction calibration, constructing a multi-dimensional evaluation system that offers a complete reference paradigm for developing clinical prediction models in hepatology. Furthermore, the core prognostic markers and their action patterns identified in this study can support the severity stratification of PBC patients, and the optimal Extreme Random Tree model enables rapid prognostic assessment, providing solid quantitative evidence for clinicians to formulate individualized intervention strategies. Figure 1 presents the technical flowchart of this study.

The remainder of this paper is organized as follows: Section 2 details data sources, missing value handling, feature engineering, model construction, and performance evaluation metrics; Section 3 presents the predictive performance of each model on training, testing, and external validation datasets, including fundamental classification metrics and visualization analysis results. SHAP-based interpretability analysis reveals key prognostic features and their underlying mechanisms, followed by in-depth discussion integrating clinical and pathological knowledge; Section 4 summarizes the study conclusions, limitations, and future directions for clinical translation.

## 2 Materials and methods

### 2.1 Data sources

This paper used a multicenter real-world clinical dataset of primary biliary cholangitis (PBC) retrieved from Kaggle (2023). All enrolled patients met the international unified diagnostic criteria for PBC. Patients complicated with hepatocellular carcinoma, end-stage renal failure and other life-threatening severe comorbidities were excluded. Standard inclusion criteria: (1) clinical diagnosis of definite PBC based on serology, liver biochemistry and cholangiography/liver histology; (2) complete baseline core laboratory and clinical symptom records at initial diagnosis; (3) standardized UDCA or D-penicillamine treatment with documented long-term follow-up outcome. Exclusion criteria: patients complicated with hepatocellular carcinoma, end-stage renal failure, decompensated heart failure or other life-threatening terminal severe comorbidities that independently drive poor survival prognosis; patients with incomplete key follow-up outcome labels; patients with missing core prognostic laboratory indicators without recoverable records.

The total raw dataset was randomly partitioned into two fully independent, non-overlapping cohorts with zero shared patient samples before any preprocessing or modeling: an internal cohort containing 7905 patients and an independent external validation cohort containing 2372 patients. The external validation cohort served as a fully held-out out-of-sample dataset for cross-dataset external validation and was never involved in training, testing, hyperparameter tuning or feature selection procedures of any model. Identical stratified missing value imputation pipelines were applied to both cohorts using only statistics derived from the internal training set to avoid data leakage and guarantee consistent data quality across cohorts. The dataset covers multi-dimensional information including demographic characteristics (age, sex), treatment regimens (drug type, n_days), clinical manifestations (ascites, hepatomegaly, spider angioma, edema), laboratory biomarkers (bilirubin, albumin, prothrombin, copper, alkaline phosphatase, platelets, etc.) and disease staging. The variable n_days represents the number of days from a patient’s initial PBC diagnosis to the earliest occurrence among death, liver transplantation, or the study’s analysis cutoff time. The prediction task of this study was to classify patients into two binary follow-up outcomes: stable remission or disease progression after standardized long-term treatment, the judgment criteria for the outcome labels were formulated in accordance with the clinical practice guidelines for PBC: (1) Stable remission (status = 1): Sustained complete biochemical response to standard therapy throughout the entire follow-up period; no new-onset cirrhosis or portal hypertensive complications (ascites, variceal bleeding), no liver transplantation, and no PBC-attributable mortality during follow-up; no progression in liver fibrosis stage. (2) Disease progression (status = 0): Occurrence of any PBC-related adverse event during follow-up, including aggravated liver fibrosis stage, new-onset cirrhosis, ascites, esophagogastric variceal bleeding, hepatic encephalopathy, liver transplantation, or liver-related mortality caused by primary biliary cholangitis.

### 2.2 Data preprocessing

Statistical analysis and handling of missing values in the dataset variables are crucial for reducing the risk of introducing systematic bias and for subsequent model evaluation. For categorical variables such as sex, drug, ascites, hepatomegaly, spiders and edema, mode imputation (Song et al., 2024) was applied. This approach aims to preserve the original category distribution while filling missing values, thereby avoiding the introduction of new categorical bias. To prevent data leakage, imputation was performed only on the training set. Second, for continuous clinical indicators like bilirubin, cholesterol, albumin, triglycerides, platelets, and prothrombin, a median imputation (Nakagawa and Sozu, 2024) strategy is employed. This is because these medical indicators often exhibit skewed distributions in PBC patients, and the median demonstrates greater robustness against extreme outliers compared to the mean. This approach reflects a stratified missing value handling strategy based on data type and clinical characteristics. It preserves data integrity while minimizing disruption to the true physiological information structure, providing more stable and reasonable input features for subsequent model training. For continuous variables like copper, alkaline phosphatase (Alk_Phos), SGOT, follow-up survival days (n_days), and age. These indicators typically exhibit strong clinical correlations, and simple statistical imputation may disrupt their intrinsic relationships. Therefore, the K-Nearest Neighbors (KNN) imputation (Zhang, 2012) method was employed. This approach estimates missing values by identifying the most similar patient samples in the feature space, thereby better preserving the structural information among multiple variables. Finally, this work removed the ID column, which serves only for sample identification and lacks predictive significance, to prevent irrelevant features from interfering with model learning.

### 2.3 Feature engineering

Through a series of creative steps—data cleaning, feature transformation, new feature construction, and important feature selection—feature engineering transforms raw data into information that better reveals the essence of the problem (Shil et al., 2026). This directly enhances a model’s predictive accuracy, generalization capability, and interpretability. As a crucial step in data preprocessing, it enables categorical variables (e.g., sex, drug, ascites) to be input into machine learning models for training. Different categorical features require distinct encoding strategies. Considering age exhibits a distinct nonlinear inflection point affecting PBC survival prognosis (e.g., accelerated disease risk around age 50 and significantly elevated mortality risk above 70), using raw continuous values allows tree models to capture some nonlinearity. However, binning yields clinically more meaningful boundaries and enables models to better identify critical thresholds. This study converted raw age (in days) to years and discretized it into four integer categories based on age brackets (0: <50 years, 1: 50–59 years, 2: 60–69 years, 3: ≥70 years), generating the new feature column “age_group”. This work also calculated the bilirubin-to-albumin ratio (bilirubin_albumin_ratio) and computed a weighted composite symptom score (symptom_score, range 0–6 points) based on four clinical signs: ascites, hepatomegaly, spiders, and edema) to derive a weighted symptom score (symptom_score, range 0–6 points, where ascites and edema each count as 2 points, and hepatomegaly and spiders each count as 1 point). The bilirubin_albumin_ratio essentially serves as a core simplified version of the ALBI score, effectively capturing severe imbalances in hepatic metabolic and synthetic functions. The symptom composite score integrates multiple clinical signs into a single weighted numerical gradient, outperforming standalone one-hot encoding by enabling tree models to better capture the nonlinear impact of overall disease severity. Considering that most mainstream machine learning models require both input features and target variables to be numerical, all categorical variables were converted to integer labels in this study.

**FIGURE 2:**
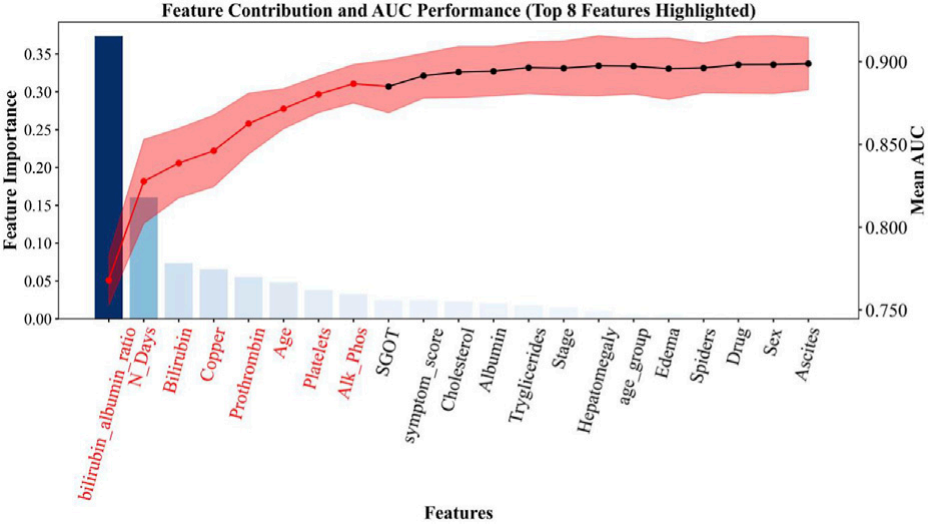
Recursive feature selection results based on random forest algorithm for PBC prognostic prediction.

Feature selection plays a critical role prior to model training, as it effectively reduces noise and mitigates overfitting by eliminating irrelevant and redundant features. Recursive feature selection based on random forests (Boughorbel and Al-Ali, 2015) is adopted for feature screening on the training set (Figure 2). This algorithm identifies key features by creating shadow features (random copies of features) and evaluating the importance of original features using the random forest algorithm. Ultimately, eight acceptable variables were selected: bilirubin_albumin_ratio, n_days, bilirubin, copper, prothrombin, age, platelets, Alk_Phos.

### 2.4 Machine learning models

This paper utilizes Python libraries including pandas, numpy, matplotlib, and sklearn. The eight selected feature variables are input into six distinct machine learning models: Logistic Regression (LR) (Kathiresan et al., 2023), Random Forest (RF) (Kaur and Garg, 2024), Extreme Tree (ET) (Dalleau et al., 2018), Extreme Gradient Boosting (XGBoost) (Chen et al., 2016), Lightweight Gradient Boosting Machine (LightGBM) (Meng, 2017), and Multi-Layer Perceptron (MLP) (Sivakumar and Desai, 2002). Logistic regression is a probabilistic discriminative model based on generalized linear models. It maps linear combinations to the [0,1] probability range via the sigmoid function, modeling the relationship between features and labels. Random forest is an ensemble decision tree model based on bagging. It constructs multiple independent decision trees by randomly sampling training samples and features, outputting the final prediction result through majority voting. Extreme Random Trees further enhance randomness by using the entire dataset for node splitting and randomly selecting splitting thresholds. XGBoost achieves precise classification by using the negative gradient of the loss function as residuals for current fitting. It reduces overfitting through quadratic Taylor series expansion of the loss function and adds regularization terms to balance loss reduction with model complexity. LightGBM is a lightweight refinement of gradient boosting, employing gradient-based one-sided sampling and mutually exclusive feature bundling to reduce computational and storage overhead. It enhances fitting performance through a leaf-node priority growth strategy. The Multi-Layer Perceptron takes raw features as input, progressively learning higher-order abstract features through hidden layers. The output layer employs a Softmax activation function to map outputs to posterior probability distributions across categories. It minimizes cross-entropy loss via backpropagation and gradient descent optimization to determine sample classifications.

In this study, the complete internal dataset was randomly split into a 70% training subset and a 30% test subset. All missing value imputation, feature standardization, SMOTE oversampling, feature selection, and hyperparameter tuning procedures were exclusively performed on the training set, which effectively avoids the overestimation of AUC values that would occur if these operations were carried out prior to dataset partitioning. Given the wide variation in the value ranges of different features, min-max normalization was applied to all variables using only the minimum and maximum values derived from the training set. Meanwhile, the SMOTE algorithm was adopted to generate synthetic minority samples and alleviate class imbalance within the training dataset. For each machine learning model, a grid search algorithm was implemented to identify the optimal hyperparameters by maximizing the AUC of the ROC curve on the training set, ensuring stable predictive performance on both the test set and independent external validation cohort. Five-fold cross-validation was applied to all models to enhance the robustness of the experimental results (Kalita et al., 2026).

This study implemented all models using Python 3.8, with core libraries including pandas 1.4.2, numpy 1.22.3, scikit-learn 1.0.2, xgboost 1.5.2 and lightgbm 3.3.2. The detailed hyperparameter search range and final optimal parameters for each model are as follows: (1) LR: Search range: penalty = [l2], C = [0.1, 1, 10], solver = [lbfgs, saga]. Optimal parameters: penalty = l2, C = 0.1, solver = lbfgs. (2) RF: Search range: n_estimators = [50, 100, 200], max_depth = [10, 20, None], min_samples_split = [2, 5, 10]. Optimal parameters: n_estimators = 200, max_depth = 10, min_samples_split = 10. (3) ET: Search range: n_estimators = [50, 100, 200], min_samples_split = [2, 5, 10], max_features = [‘sqrt’, ‘log2’], criterion = ['gini’, ‘entropy']. Optimal parameters: n_estimators = 200, criterion = gini, max_features = sqrt, min_samples_split = 10. (4) XGBoost: Search range: learning_rate = [0.01, 0.1, 0.3], max_depth = [3, 5, 7], n_estimators = [100, 200]. Optimal parameters: learning_rate = 0.1, max_depth = 3, n_estimators = 200. (5) LightGBM: Search range: learning_rate = [0.01, 0.1, 0.3], num_leaves = [31, 50, 100], n_estimators = [100, 200]. Optimal parameters: learning_rate = 0.1, num_leaves = 31, n_estimators = 200. (6) MLP: Search range: hidden_layer_sizes = [(50) (100) (100, 50)], learning_rate_init = [0.001, 0.01], max_iter = [200, 300]. Optimal parameters: hidden_layer_sizes = (100, 50), learning_rate_init = 0.01, max_iter = 200.

To objectively evaluate the classification performance and generalization capability of each predictive model, this study employs a multidimensional evaluation metric for comprehensive quantitative analysis. Overall classification effectiveness is measured using accuracy, precision, sensitivity, specificity, and F1 score as foundational metrics; Model discrimination and ranking capabilities were measured using AUC and Average Precision (AP). To visually illustrate model performance differences, ROC curves, PR curves, decision curve analysis, and calibration curves were plotted. This enabled a comprehensive and rigorous evaluation of model performance across classification discrimination, imbalanced data adaptability, clinical decision value, and prediction calibration.

### 2.5 SHAP explainability analysis

To delve into the decision-making mechanisms of predictive models and clarify the influence of key features on classification outcomes, this study employs SHAP values for model interpretability analysis. As a state-of-the-art method in machine learning model interpretation, SHAP draws its theoretical foundation from the Shapley value in game theory. Its core concept treats the model’s overall prediction outcome as a “game result” jointly produced by all input features. By quantifying the marginal contribution of each feature (i.e., game participant) to the final prediction, SHAP achieves interpretability and traceability of the model’s decision-making process. Compared to traditional feature importance evaluation methods, SHAP values offer dual advantages of global consistency and local interpretability. At the global level, the mean SHAP value quantifies the overall influence weight of each feature on model prediction performance, clearly identifying key discriminative features. At the local level, it provides an intuitive visualization for individual samples, showing the positive or negative contribution of each feature to the prediction outcome for that specific sample, thereby clearly explaining the model’s classification logic for particular instances (Lundberg et al., 2020).

### 2.6 Statistical analysis

Python was used for grouping and statistical analysis. Continuous variables are presented as median (interquartile range), while categorical data are shown as counts and corresponding percentages. The Wilcoxon signed-rank test was used to compare continuous variables, and the chi-square test was used to compare categorical variables, analyzing differences in demographic and clinical characteristics between the PBC stable remission group (status = 1) and the disease progression group (status = 0).

## 3 Results and discussion

### 3.1 Patient baseline characteristics

Among the 7905 PBC patients included in this analysis, 5240 achieved disease remission with treatment, while 2665 experienced disease progression. Baseline characteristics for both groups are shown in Table 1. Differences in all indicators except the drug were statistically significant between the two groups (all P < 0.001).

**TABLE 1:** Clinical characteristics of patients (n = 7905).

Variables	PBC		
	C (n = 5240)	D (n = 2665)	P-value
N Days (day)	2176.00 (1447.00–2891.00)	1216.00 (769.00–2297.00)	<0.001
Age (day)	17889.00 (15000.50–20459.00)	19256.00 (16898.00–22336.00)	<0.001
Bilirubin (mg/dL)	0.90 (0.60–1.30)	3.20 (1.30–6.40)	<0.001
Cholesterol (mg/dL)	293.00 (248.00-360.00)	331.00 (248.00–448.00)	<0.001
Albumin (mg/dL)	3.61 (3.43-3.80)	3.45 (3.20-3.67)	<0.001
Copper (μg/V day)	51.00 (34.00–75.00)	101.00 (60.00–159.00)	<0.001
Alkaline Phosphatase (U/mL)	1098.00 (766.00-1636.00)	1580.00 (1009.00-2374.00)	<0.001
SGOT (U/mL)	94.55 (71.30-127.71)	130.20 (102.30–164.30)	<0.001
Triglycerides (mg/dL)	101.00 (80.00-130.00)	114.00 (90.00-154.00)	<0.001
Platelets (ml/L)	271.00 (226.00-324.00)	228.00 (181.00–297.00)	<0.001
Prothrombin time (s)	10.40 (9.90-10.80)	11.00 (10.50-11.50)	<0.001
Drug			0.227
Placebo	2684 (51.2%)	1326 (49.8%)	
D-penicillamine	2556 (48.8%)	1339 (50.2%)	
Se**x**			<0.001
Female	4986 (95.2%)	2350 (88.2%)	
Male	254 (4.8%)	315 (11.8%)	
Ascites			<0.001
N	5209 (99.4%)	2316 (86.9%)	
Y	31 (0.6%)	349 (13.1%)	
Hepatomegaly			<0.001
N	3283 (62.7%)	580 (21.8%)	
Y	1957 (37.3%)	2085 (78.2%)	
Spiders			<0.001
N	4465 (85.2%)	1501 (56.3%)	
Y	775 (14.8%)	1164 (43.7%)	
Edema			<0.001
N	5104 (97.4%)	2057 (77.2%)	
S	126 (2.4%)	273 (10.2%)	
Y	10 (0.2%)	335 (12.6%)	
Stage			<0.001
1	358 (6.8%)	39 (1.5%)	
2	1340 (25.6%)	312 (11.7%)	
3	2411 (46.0%)	742 (27.8%)	
4	1131 (21.6%)	1572 (59.0%)	

### 3.2 Multi-model ensemble classification analysis

**FIGURE 3:**
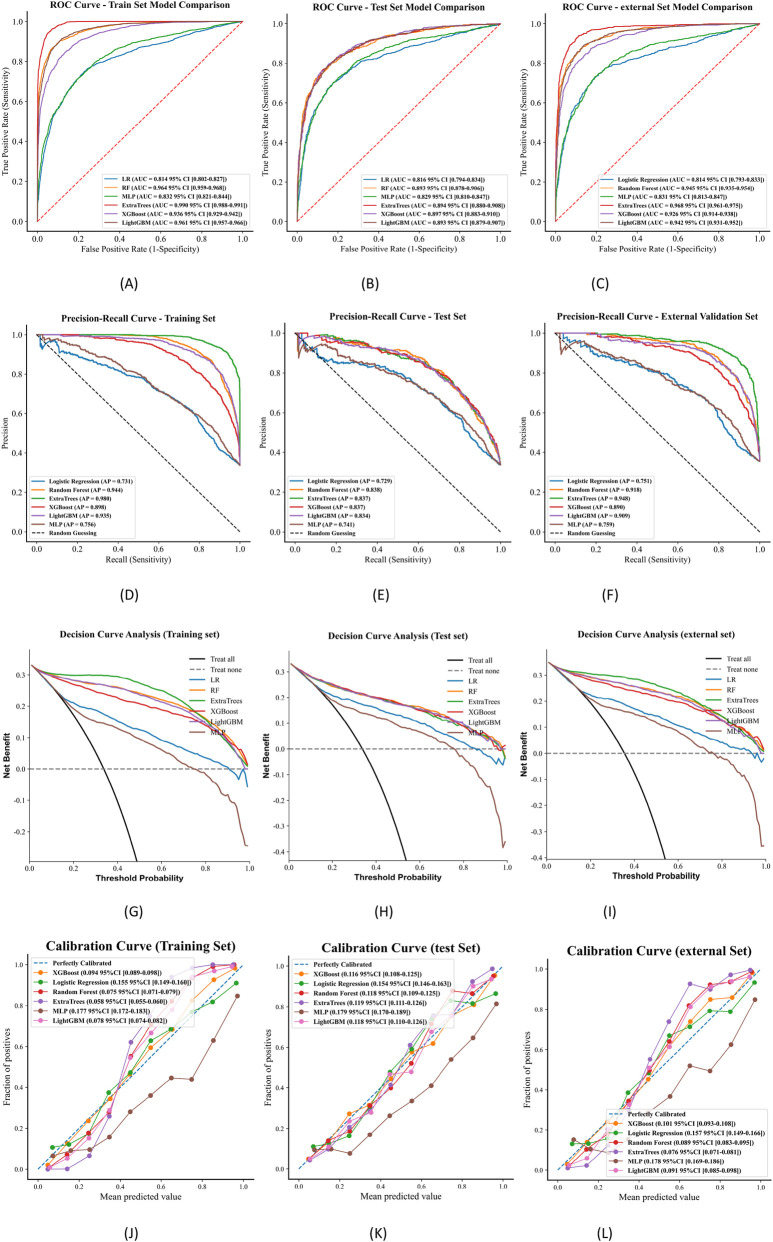
Predictive performance of machine learning models. **(A)** ROC curve for the training set; **(B)** ROC curve for the test set; **(C)** ROC curve for external validation set; **(D)** PR curve for training set; **(E)** PR curve for test set; **(F)** PR curve for external validation set; **(G)** DCA curve for training set; **(H)** DCA curve for test set; **(I)** DCA curve for external validation set; **(J)** CC curve for training set; **(K)** CC curve for test set; **(L)** CC curve for external validation set.

This study comprehensively quantifies and visually analyzes the classification performance of six machine learning models-across three datasets: training, testing, and external validation. Using accuracy, sensitivity, precision, specificity, and F1 score as fundamental classification metrics. This work also quantified model discrimination and ranking capabilities using AUC and AP (Table 2). Additionally, ROC curves, PR curves, DCA, and CC were plotted to achieve a comprehensive comparison of multi-model performance across four dimensions: classification efficacy, imbalanced data adaptability, clinical decision value, and predictive probability calibration (Figure 3).

**TABLE 2:** Performance metrics results for each model.

Dataset	Model	LR (95% CI)	RF (95% CI)	ET (95% CI)	XGBoost (95% CI)	LightGBM (95% CI)	MLP (95% CI)
Training Set	Accuracy	0.7875 [0.7770, 0.7979]	0.9038 [0.8955, 0.9111]	0.9360 [0.9292, 0.9423]	0.8708 [0.8612, 0.8787]	0.8988 [0.8903, 0.9064]	0.7587 [0.7475, 0.7697]
	Sensitivity	0.5421 [0.5198, 0.5662]	0.7769 [0.7578, 0.7971]	0.8520 [0.8353, 0.8692]	0.7373 [0.7168, 0.7568]	0.7898 [0.7723, 0.8090]	0.7539 [0.7332, 0.7740]
	Precision	0.7584 [0.7356, 0.7810]	0.9259 [0.9132, 0.9380]	0.9532 [0.9427, 0.9630]	0.8594 [0.8429, 0.8758]	0.8976 [0.8831, 0.9121]	0.6161 [0.5959, 0.6367]
	Specificity	0.9122 [0.9032, 0.9207]	0.9684 [0.9627, 0.9739]	0.9787 [0.9740, 0.9833]	0.9387 [0.9310, 0.9457]	0.9542 [0.9473, 0.9610]	0.7612 [0.7469, 0.7748]
	F1_Score	0.6323 [0.6119, 0.6531]	0.8449 [0.8332, 0.8580]	0.8998 [0.8893, 0.9105]	0.7937 [0.7785, 0.8074]	0.8403 [0.8274, 0.8534]	0.6781 [0.6608, 0.6950]
	AUC	0.8144 [0.8020, 0.8270]	0.9637 [0.9590, 0.9680]	0.9898 [0.9880, 0.9910]	0.9355 [0.9290, 0.9420]	0.9615 [0.9570, 0.9660]	0.8324 [0.8210, 0.8440]
Test Set	Accuracy	0.7930 [0.7774, 0.8086]	0.8423 [0.8267, 0.8567]	0.8415 [0.8267, 0.8558]	0.8398 [0.8255, 0.8546]	0.8352 [0.8204, 0.8503]	0.7555 [0.7386, 0.7728]
	Sensitivity	0.5662 [0.5339, 0.6008]	0.6850 [0.6526, 0.7184]	0.6925 [0.6622, 0.7255]	0.7075 [0.6771, 0.7399]	0.7000 [0.6700, 0.7312]	0.7612 [0.7332, 0.7915]
	Precision	0.7588 [0.7251, 0.7939]	0.8179 [0.7889, 0.8464]	0.8099 [0.7799, 0.8387]	0.7949 [0.7649, 0.8230]	0.7876 [0.7571, 0.8201]	0.6102 [0.5808, 0.6408]
	Specificity	0.9084 [0.8938, 0.9230]	0.9224 [0.9096, 0.9345]	0.9173 [0.9032, 0.9304]	0.9071 [0.8927, 0.9203]	0.9039 [0.8891, 0.9182]	0.7525 [0.7318, 0.7742]
	F1_Score	0.6485 [0.6208, 0.6761]	0.7456 [0.7208, 0.7701]	0.7466 [0.7220, 0.7716]	0.7487 [0.7250, 0.7725]	0.7412 [0.7172, 0.7662]	0.6774 [0.6521, 0.7010]
	AUC	0.8157 [0.7940, 0.8340]	0.8926 [0.8780, 0.9060]	0.8939 [0.8800, 0.9080]	0.8968 [0.8830, 0.9100]	0.8935 [0.8790, 0.9070]	0.8292 [0.8100, 0.8470]
External Validation Set	Accuracy	0.7879 [0.7728, 0.8052]	0.8820 [0.8689, 0.8955]	0.9068 [0.8950, 0.9182]	0.8651 [0.8516, 0.8794]	0.8794 [0.8672, 0.8933]	0.7631 [0.7462, 0.7808]
	Sensitivity	0.5582 [0.5237, 0.5914]	0.7542 [0.7248, 0.7840]	0.8064 [0.7803, 0.8335]	0.7494 [0.7197, 0.7797]	0.7732 [0.7439, 0.8030]	0.7672 [0.7399, 0.7952]
	Precision	0.7820 [0.7496, 0.8168]	0.8969 [0.8750, 0.9198]	0.9213 [0.9008, 0.9399]	0.8527 [0.8276, 0.8794]	0.8727 [0.8475, 0.8973]	0.6383 [0.6069, 0.6680]
	Specificity	0.9144 [0.9014, 0.9284]	0.9523 [0.9413, 0.9625]	0.9621 [0.9524, 0.9710]	0.9288 [0.9154, 0.9415]	0.9379 [0.9251, 0.9499]	0.7608 [0.7409, 0.7819]
	F1_Score	0.6514 [0.6209, 0.6812]	0.8194 [0.7968, 0.8416]	0.8600 [0.8406, 0.8781]	0.7977 [0.7746, 0.8200]	0.8199 [0.7987, 0.8412]	0.6969 [0.6703, 0.7213]
	AUC	0.8137 [0.7930, 0.8330]	0.9454 [0.9350, 0.9540]	0.9681 [0.9610, 0.9750]	0.9263 [0.9140, 0.9380]	0.9418 [0.9310, 0.9520]	0.8305 [0.8130, 0.8470]

Within the training set, ensemble tree models demonstrated significantly superior overall performance compared to traditional linear models and shallow neural network models. Among these, the ET model achieved optimal metrics across all indicators, with AUC and AP reaching 0.9898 and 0.9804 respectively, while accuracy and F1 score reached 0.9360 and 0.8998. RF and LightGBM models demonstrated comparable performance, both achieving AUC values above 0.96, indicating excellent fitting capabilities. The XGBoost model recorded an AUC of 0.9355, slightly lower than the aforementioned ensemble tree models. In contrast, LR and MLP models exhibited relatively weaker performance, with AUC values of 0.8144 and 0.8324, respectively, and all fundamental classification metrics significantly below those of the ensemble tree models. ROC curve results indicate that all six models’ curves significantly deviate from the random guessing line on the training set. Ensemble tree models demonstrate a pronounced advantage in area under the curve, further validating their robust classification capabilities on the training data. The PR curve results align with the AUC trend. The ET model achieved the highest AP value. The ensemble tree model maintained high precision across the high recall range, whereas the LR and MLP models exhibited a noticeable decline in precision as recall increased, indicating poorer adaptability to imbalanced data.

The test set serves as an initial validation of model generalization capability. While all models exhibit varying degrees of performance decline compared to the training set, the performance ranking remains largely unchanged, with the ensemble tree model retaining its performance advantage. XGBoost demonstrated optimal performance on the test set with an AUC of 0.8968 (95% CI [0.883–0.910]). ET, RF, and LightGBM exhibited comparable performance with AUC values of 0.8939, 0.8926, and 0.8935, respectively, with highly overlapping 95% confidence intervals. This indicates no significant differences in classification efficacy among the four ensemble tree models on the test set. The LR and MLP models achieved AUC values of 0.8157 and 0.8292, respectively, which were largely consistent with their training set performance, showing no obvious overfitting. However, their overall discriminative capability remained at a relatively low level. DCA curve results revealed that all machine learning models demonstrated higher net benefit than the “treat all” and “treat none” strategies across both training and test sets. Among these, ensemble tree models consistently exhibited significantly higher net benefit than LR and MLP models across the entire threshold probability range. Specifically, RF and ET models achieved peak net benefit within the clinically critical threshold range of 0.2–0.8, indicating superior clinical decision-guidance value. Calibration curve results indicate that the ET model achieved optimal alignment between predicted and actual probabilities in both training and testing datasets, with calibration errors of 0.058 (95% CI [0.055–0.060]) and 0.119 (95% CI [0.111–0.126]), respectively.; RF and LightGBM models exhibited similar calibration errors at relatively low levels; XGBoost model calibration performance was slightly inferior to the aforementioned models, while LR and MLP models showed significantly higher calibration errors. Notably, the MLP model reached a calibration error of 0.179 (95% CI [0.170–0.189]) in the test set, indicating substantial deviation between predicted and actual probabilities.

The external validation set, serving as independent cross-center data, provides the core validation basis for the model’s practical application value. Results show that the ET model demonstrates optimal generalization performance on the external validation set, achieving AUC and AP of 0.9681 and 0.9479, respectively. Accuracy and F1 score also reached 0.9068 and 0.8600, respectively, showing a performance rebound compared to the test set and exhibiting strong cross-dataset adaptability. The RF and LightGBM models maintained excellent performance, achieving AUC values of 0.9454 and 0.9418 respectively, with all fundamental classification metrics remaining at high levels. The XGBoost model recorded an AUC of 0.9263, slightly lower than the top three but still significantly outperforming traditional models. The LR and MLP models exhibited stable performance on the external validation set, achieving AUC values of 0.8137 and 0.8305 respectively. Their performance aligned with that on the training and test sets, showing no signs of overfitting or underfitting. However, their overall classification efficacy still lagged significantly behind ensemble tree models. The PR curve for the external validation set shows that the ET model maintains precision above 0.8 across the entire recall range, achieving an AP value of 0.9479. Its adaptability to imbalanced data is the best among all models. DCA curve results indicate that the clinical net benefit of the ensemble tree model remains significantly higher than that of the LR and MLP models in the external validation set. Among these, the ET and RF models consistently maintain positive net benefits within the 0.1–0.9 threshold range, significantly outperforming other models and demonstrating outstanding clinical application value. Calibration curve results demonstrate improved calibration performance across all models in the external validation set. The calibration error of the ET model further decreased to 0.076 (95% CI [0.071–0.081]), while the RF model reached 0.089 (95% CI [0.083–0.095]). with both models exhibiting optimal alignment between predicted probabilities and actual clinical outcomes. In contrast, the LR and MLP models maintained high calibration errors, indicating poor reliability of their predicted probabilities.

Overall, ensemble tree models (ET, RF, LightGBM, XGBoost) demonstrated significant performance advantages in predicting prognosis for PBC patients, exhibiting excellent classification discrimination, generalization capability, and clinical decision value. Among these, the ET model achieved optimal performance on both the training and external validation sets, while the RF model showed comparable performance with greater stability. Traditional linear models (LR) and shallow neural network models (MLP), while not exhibiting obvious overfitting, demonstrated significantly lower overall classification efficacy, imbalanced data adaptability, and prediction probability calibration compared to ensemble tree models, failing to meet the demands of clinical precision prediction.

### 3.3 Model interpretability analysis

#### 3.3.1 Global feature importance analysis

**FIGURE 4:**
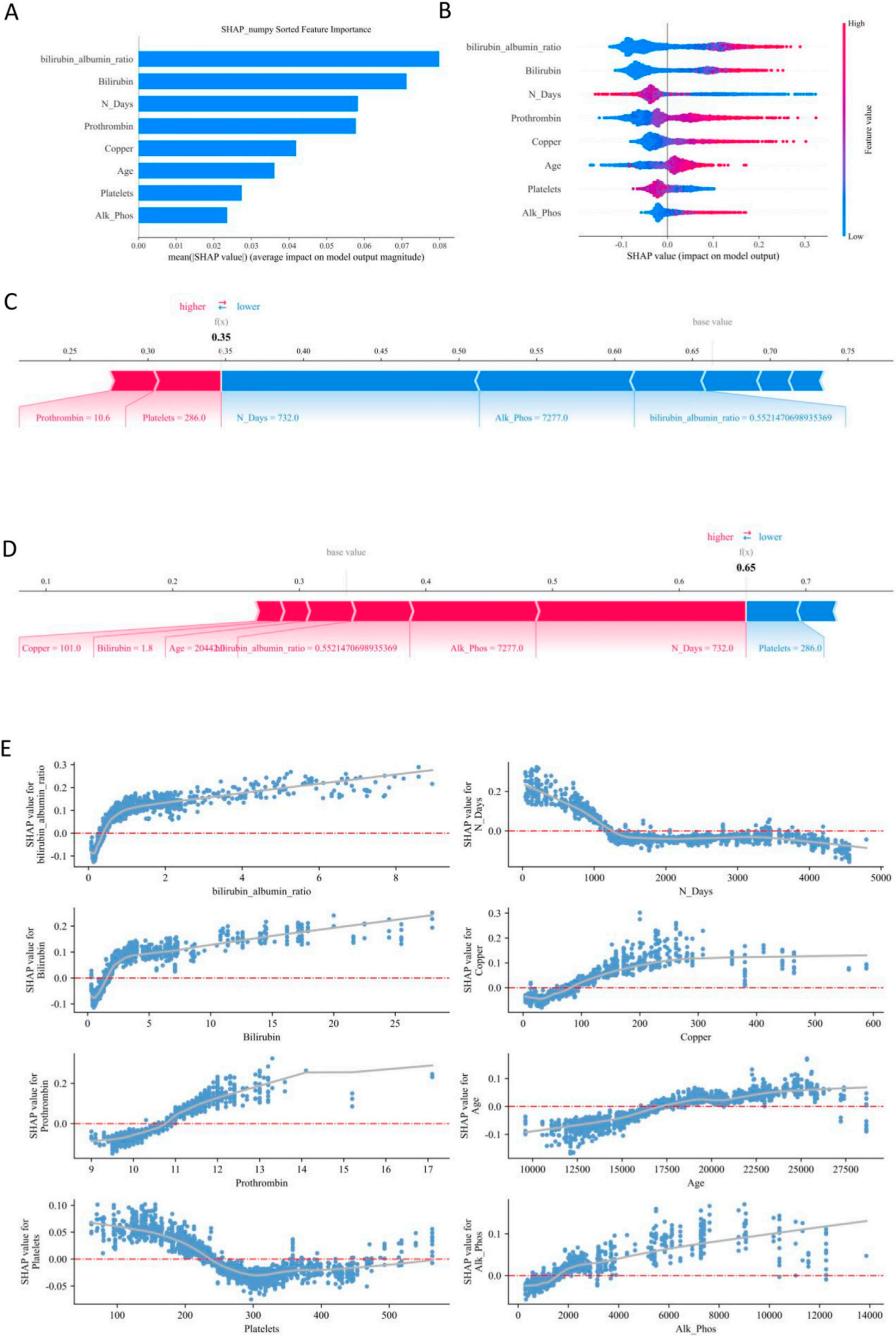
SHAP results. **(A)** SHAP overall feature importance ranking plot; **(B)** Global interpretability analysis plot; **(C)** SHAP force plot for the first sample in class 0; **(D)** SHAP force plot for the first sample in class 1; **(E)** SHAP dependency plots for each feature.

The overall feature importance ranking based on SHAP values (Figure 4A) indicates that the bilirubin-to-albumin ratio (bilirubin_albumin_ratio) has the greatest impact on model prediction output, making it the core feature for predicting prognosis in PBC patients. followed by bilirubin, n_days, prothrombin, copper, and age. Platelets and Alk_Phos exhibited relatively weaker influence. This ranking of feature importance shows descriptive consistency with established clinical-pathological mechanisms. Metabolic and synthetic function indicators of the liver (bilirubin, albumin) serve as core clinical markers reflecting PBC disease severity. Their ratio directly reflects hepatocyte injury and hepatic compensatory status, forming the key basis for model prediction. This consistency validates the rationality of feature selection and engineering in this study.

#### 3.3.2 Analysis of feature global influence patterns

The model’s global interpretability analysis (Figure 4B) visually demonstrates the trend of how changes in core feature values influence model predictions. SHAP values for the bilirubin_albumin_ratio, bilirubin, and prothrombin show a significant upward trend as these indicators increase, indicating that higher values of these indicators are associated with a higher probability of the model predicting a poor prognosis for PBC patients. Bilirubin_albumin_ratio, bilirubin, and prothrombin exhibit a significant upward trend in SHAP values as their respective values increase, indicating that higher values of these indicators correlate with a greater probability of the model predicting poor prognosis in PBC patients. SHAP values for n_days, copper and platelets decrease as indicator values increase, indicating that longer follow-up survival time and higher platelet or copper levels correlate with lower predicted probability of poor prognosis. The SHAP value for the age indicator exhibited a nonlinear change, significantly increasing in the elderly age group (≥70 years), suggesting advanced age as a risk factor for poor prognosis in PBC patients. These feature patterns are consistent with existing clinical knowledge that elevated bilirubin and coagulation abnormalities indicate the progression of liver failure, and also align with the well-recognized conclusion that elderly patients have poor hepatic reserve function and a high incidence of complications.

#### 3.3.3 Local decision logic analysis for individual samples

To decipher the model’s predictive decision-making process for individual samples, typical samples were selected to generate SHAP plots (Figures 4C,D), quantifying the positive/negative contributions of each feature to the prediction outcome at the local level. Results indicate that the model’s prediction for each sample arises from the combined effects of multiple features. Taking the category prediction for Sample 0 as an example (Figure 4C), the bilirubin_albumin_ratio (0.552) and Alk_Phos (7277.0) were the primary positive contributors, significantly increasing the sample’s prediction score. Conversely, n_days (732.0) and platelets (286.0) were the primary negative contributors, exerting an inhibitory effect on the prediction score. Ultimately, the model integrates the marginal contributions of each feature to derive the prediction result for this sample. The SHAP plots for Sample 1 category prediction (Figure 4D) exhibit similar feature contribution patterns, with the core feature bilirubin_albumin_ratio remaining the most significant positive influence factor, validating the critical role of core features in single-sample prediction. Concurrently, the SHAP plot clearly displays the model’s base prediction value, with SHAP values for each feature fluctuating positively or negatively around this baseline. This enables traceability and interpretability of the single-sample prediction outcome.

#### 3.3.4 Feature dependency analysis

The SHAP dependency plots for each feature (Figure 4E) further reveal quantitative associations between feature values and SHAP values, clarifying the critical ranges and trends of feature influence on model predictions. Within the 0–0.6 range for the bilirubin_albumin_ratio, SHAP values exhibit a rapid upward trend as the ratio increases. Beyond 0.6, the rate of SHAP value increase slows, indicating that 0.6 is a critical threshold for the model to identify abnormal liver function. For bilirubin levels between 0 and 15 μmol/L, SHAP values exhibit a linear increase. This range corresponds to the clinically significant interval for elevated bilirubin, aligning closely with the model’s recognition patterns.

## 4 Summary

### 4.1 Conclusion

Based on multicenter clinical data, this study systematically constructed and validated six machine learning models to achieve precise prognosis prediction for PBC patients. The ensemble tree model demonstrated significant performance advantages in PBC prognosis prediction tasks. Compared with traditional linear logistic regression models and shallow neural network multi-layer perceptron models, it exhibited superior classification discrimination capability, generalization ability, adaptability to imbalanced data, and prediction probability calibration. Among these, the extreme random tree model demonstrated optimal predictive performance on both the training set and external validation set, while the random forest model exhibited superior stability. Both model types are suitable for clinical prognosis assessment in PBC patients.

Machine learning models analyzed via the SHAP method exhibit strong global and local interpretability. They can quantify the overall influence weight of each feature on model predictions while also tracing the predictive decision logic for individual samples. The bilirubin_albumin_ratio identified as a core prognostic feature essentially serves as a comprehensive quantitative indicator of hepatic metabolic and synthetic function. Elevated levels reflect both impaired hepatocyte metabolism and excretion of bilirubin and reduced albumin synthesis capacity. Together, these changes characterize hepatic decompensation, aligning closely with the core pathological alterations in PBC progression. This finding also validates the rationale behind the core logic of the ALBI score in PBC prognosis assessment. The prioritization of prothrombin, copper, and platelets shows descriptive alignment with clinical understanding: coagulation abnormalities indicate hepatic synthetic failure, thrombocytopenia reflects liver fibrosis and portal hypertension, and elevated copper correlates with cholestasis. This demonstrates that the model’s data-driven decision patterns do not contradict known PBC pathophysiology, significantly enhancing its credibility and acceptability in clinical practice. Concurrently, the SHAP dependency map revealed critical thresholds such as a bilirubin_albumin_ratio of 0.6 and an age of 54.8 years. These provide novel reference points for establishing quantitative standards for PBC disease stratification in clinical practice, enabling direct translation of the model’s predictions into clinically actionable assessment metrics.

The proposed explainable machine learning models have strong practical value for real-world PBC clinical management. First, the core input features of the model are routine laboratory indicators and basic clinical signs that are universally available in all levels of medical institutions, without relying on expensive imaging, genetic testing or professional psychological scales. It can be applied to tertiary specialized liver disease centers, general hospitals and even primary medical institutions for rapid preliminary screening. Second, the model realizes binary classification of disease remission and progression, and combined with SHAP interpretation results, clinicians can directly obtain the contribution degree of each indicator to individual patient prognosis, so as to quickly identify high-risk factors such as abnormal bilirubin-albumin ratio and prolonged prothrombin time, and formulate targeted intervention schemes. In real clinical workflow, this model can be embedded into hospital electronic medical record systems in the form of lightweight plug-ins or web tools. After clinicians input patients’ age, laboratory results and clinical manifestations, the system can automatically output prognosis prediction results and corresponding feature interpretation reports within 1 s, realizing bedside rapid assessment. For high-risk patients predicted to have disease progression, clinicians can timely adjust treatment schemes, strengthen follow-up frequency and monitor liver function dynamically; for low-risk patients with stable remission, regular follow-up can be adopted to reduce medical burden.

### 4.2 Limitations

Although this study successfully constructed and validated an interpretable machine learning model for predicting prognosis in PBC patients, several limitations remain, indicating directions for future optimization.

1. Data completeness limitation. Although stratified missing value imputation strategies are adopted for different types of variables to reduce bias, partial missing data in the original clinical dataset may still interfere with the model’s performance. The data source is a public multicenter dataset, and unified standardized collection standards are lacking among different medical centers.
2. Incomplete selection of influencing factors. The current model only incorporates demographic data, clinical symptoms and routine laboratory indicators. Psychological status, lifestyle, long-term treatment adherence, genetic factors and other variables proven to be related to PBC progression are not included, which limits the further improvement of model predictive efficacy.
3. Single prediction form. The model only realizes binary classification of disease progression or remission, and cannot complete graded prognostic risk assessment (low/medium/high risk) or long-term survival prediction. In addition, no clinical visualized application tools are developed, which restricts the popularization and application in clinical workflow.
4. Lack of prospective cohort verification. All data in this study are retrospective clinical data. The stability and effectiveness of the model need to be further verified through prospective real-world cohorts.

### 4.3 Future research directions

1. Optimize data collection and expand feature dimensions. Establish a unified standard for PBC clinical data collection to improve data completeness. On the basis of existing indicators, supplement psychological assessment scales, lifestyle records, medication compliance and genetic detection data, build a multi-dimensional feature system, and further optimize model performance.
2. Develop graded prognostic prediction and long-term survival models. The model shows excellent internal and external generalization performance on the current dataset (external validation AUC of ET model = 0.9681). For PBC patients with similar demographic characteristics, disease staging and treatment modes in western medical institutions, the model has good generalizability and stable prediction effect. However, due to population and regional bias, the direct generalization ability of this model for Asian, African and other regional populations needs to be further verified by regional real-world data. Meanwhile, On the basis of the current binary classification model, construct multi-classification risk stratification models and survival prediction models to realize quantitative graded assessment of PBC patient prognosis (1-year, 3-year and 5-year survival risk).
3. Develop clinical visualized auxiliary tools. Combine the optimal ET model to build a lightweight web-side or terminal prediction prototype, simplify the model operation process, and form a one-stop prognostic assessment tool suitable for clinical frontline use.
4. Carry out prospective cohort validation. Cooperate with multiple clinical centers to collect prospective real-world PBC patient data, conduct external prospective verification of the model, and continuously iterate and optimize the model according to clinical feedback.

## Data Availability

The original contributions presented in the study are included in the article/Supplementary Material, further inquiries can be directed to the corresponding author.

## References

[B1] Bilal O. Hekmat A. Shahzad I. Raza A. Khan S. U. R. (2025). Boosting machine learning accuracy for cardiac disease prediction: the role of advanced feature engineering and model optimization. Rev. Socionetwork Strategies 19 (2), 1–30. 10.1007/s12626-025-00190-w

[B2] Boughorbel S. Al-Ali R. J. (2015). Variable Importance in Recursive Feature Selection with Random Forests for Mortality Prediction in ICU.

[B3] Calster B. V. Mclernon D. J. Smeden M. V. Wynants L. Steyerberg E.W. (2019). Calibration: the achilles' heel of predictive analytics and On behalf of Topic Group ‘Evaluating diagnostic tests and prediction models' of the STRATOS initiative. BMC Med. 17 (1), 137. 10.1186/s12916-019-1466-7 31842878 PMC6912996

[B4] Chen T. He T. Benesty M. (2016). Xgboost: Extreme Gradient Boosting.

[B5] Chen Q. L. Zhong R. Wang Y. Kui Y. Wen X. Huang J. (2023). The albumin-bilirubin score as a predictor of liver-related mortality in primary biliary cholangitis with compensated cirrhosis. Dig. Dis. 41 (6), 946–956. 10.1159/000531557 37321186

[B6] Corpechot C. Abenavoli L. Rabahi N. Chrétien Y. Andréani T. Johanet C. (2008). Biochemical response to ursodeoxycholic acid and long-term prognosis in primary biliary cirrhosis. Hepatology 48 (3), 871–877. 10.1002/hep.22428 18752324

[B7] Dalleau K. Couceiro M. Smail-Tabbone M. (2018). Unsupervised Extremely Randomized Trees[C]//Pacific-asia Conference on Knowledge Discovery and Data Mining. Cham: Springer.

[B8] European Association for the Study of the Liver (2017). EASL clinical practice guidelines: the diagnosis and management of patients with primary biliary cholangitis. J. Hepatol. 67 (1), 145–172. 10.1016/j.jhep.2017.03.022 28427765

[B9] Fu X. Y. Song Y. Q. Lin J. Q. Wang Y. Wu W. D. Peng J. B. (2024). Developing a prognostic model for primary biliary cholangitis based on a random survival forest model. Int. Journal Medical Sciences 21 (1), 61–69. 10.7150/ijms.88481 38164345 PMC10750344

[B10] Gao L. Wang L. Woo E. He X. Yang G. Bowlus C. (2020). Clinical management of primary biliary Cholangitis- strategies and evolving trends. Clin. Rev. Allergy and Immunol. 59 (2), 175–194. 10.1007/s12016-019-08772-7 31713023

[B11] Gerussi A. Saldanha L. O. Cazzaniga G. Verda D. Carrero Z. I. Engel B. (2025). Deep learning helps discriminate between autoimmune hepatitis and primary biliary cholangitis. JHEP Rep. 7 (2), 101198–101198. 10.1016/j.jhepr.2024.101198 39829723 PMC11741034

[B12] Jorgensen R. Angulo P. Dickson E. R. Lindor K. D. (2002). Long-term ursodiol treatment outcomes for patients with primary biliary cirrhosis. Am. J. Gastroenterology 97 (10), 2647–2650. 10.1111/j.1572-0241.2002.06043.x 12385454

[B13] Kaggle (2023). Available online at: https://www.kaggle.com/competitions/playground-series-s3e26/data (Accessed March 6, 2026).

[B14] Kalita B. K. Singh K. A. Kalita M. (2026). Fivefold cross-validation approach in evaluating the robustness of machine learning models for prediction of esophageal cancer. Indian J. Community Med. 51, S180–S188. 10.4103/ijcm.ijcm_454_24 41969709 PMC13068405

[B15] Kathiresan V. Karthik S. Prabakar D. Kavitha M. S. (2023). Logistic regression-based machine learning model for mutation classification in the discovery of precision medicine. EAI/Springer Innovations Commun. Comput., 81–92. 10.1007/978-3-031-27700-9_6

[B16] Kaur K. Garg R. (2024). Enhanced random forest approach in digital healthcare in medicine recommendation and classification. Adv. Med. Diagnosis, Treat. Care, 183–204. 10.4018/979-8-3693-1243-8.ch010

[B17] Kimura N. Takahashi K. Setsu T. Horibata Y. Kaneko Y. Miyazaki H. (2024). Development and validation of machine learning model for predicting treatment responders in patients with primary biliary cholangitis. Hepatology Research The Official Journal Jpn. Soc. Hepatology 54 (1). 10.1111/hepr.13966 37691006

[B18] Lleo A. Leung P. S. C. Hirschfield G. M. Gershwin E. M. (2020). The pathogenesis of primary biliary cholangitis: a comprehensive review. Seminars Liver Dis. 40 (01), 034–048. 10.1055/s-0039-1697617 31537031

[B19] Lundberg S. M. Erion G. Chen H. DeGrave A. Prutkin J. M. Nair B. (2020). From local explanations to global understanding with explainable AI for trees. Nat. Mach. Intell. 2, 56–67. 10.1038/s42256-019-0138-9 32607472 PMC7326367

[B20] Meng Q. (2017). Lightgbm: A Highly Efficient Gradient Boosting Decision Tree[C]//Neural Information Processing Systems. Red Hook, NY, United States: Curran Associates, Inc.

[B21] Montero A. J. (2026). Decision Curve Analysis Explained. Berlin, Germany: Diagnosis. 10.1515/dx-2025-0113 41493769

[B22] Nakagawa Y. Sozu T. (2024). Improvement of midpoint imputation for estimation of median survival time for interval-censored time-to-event data. Ther. Innovation and Regulatory Science 58 (4), 721–729. 10.1007/s43441-024-00640-7 38598082

[B23] Shil C. D. Islam R. M. Das C. S. Acharjee U. K. Islam M. M. Mahbub M. (2026). Impact of feature engineering on machine learning models for heart disease prediction across multiple data sources. Eng. Rep. 8 (2), e70566. 10.1002/eng2.70566

[B24] Shu Y. Song Y. Bai T. Pan X. Shang H. Yang L. (2021). Predictive model of ursodeoxycholic acid treatment response in primary biliary cholangitis. J. Clinical Translational Hepatology 9 (2), 187–193. 10.14218/JCTH.2020.00127 34007800 PMC8111104

[B25] Sivakumar K. Desai U. B. (2002). Image restoration using a multilayer perceptron with a multilevel sigmoidal function. IEEE Trans. Signal Process. 41 (5), 2018–2022. 10.1109/78.215329

[B26] Sohal A. Kowdley K. V. (2023). Primary biliary cholangitis: promising emerging innovative therapies and their impact on GLOBE scores. Hepat. Med. 15, 63–77. 10.2147/HMER.S361077 37312929 PMC10259525

[B27] Song J. Yang Z. Li X. (2024). Missing data imputation model for dam health monitoring based on mode decomposition and deep learning. J. Civ. Struct. Health Monit. 14 (5), 14–1124. 10.1007/s13349-024-00776-y

[B28] Vincentis D. A. Ampuero J. Terracciani F. D'Amato D. Gerussi A. Cristoferi L. (2024). Development and validation of a scoring system to predict response to obeticholic acid in primary biliary cholangitis. Clin. Gastroenterology Hepatology 22 (10), 2062–2074. 10.1016/j.cgh.2024.05.008 38782175

[B29] Wan L. L. Liang L. N. Lu S. M. (2025). Research advances in prognostic models for primary biliary cholangitis. J. Clin. Hepatol. 41 (7), 1426–1430. 10.12449/JCH250730

[B30] You H. Ma X. Efe C. Wang G. Jeong S. H. Abe K. (2022). APASL clinical practice guidance: the diagnosis and management of patients with primary biliary cholangitis. Hepatol. Int. 16 (1), 1–23. 10.1007/s12072-021-10276-6 35119627 PMC8843914

[B31] Zhang S. (2012). Nearest neighbor selection for iteratively kNN imputation. J. Syst. and Softw. 85 (11), 2541–2552. 10.1016/j.jss.2012.05.073

[B32] Zhang Y. Fan X. L. Song B. Liu Y. Chen Y. Zheng T. (2024). Noninvasive prediction of insufficient biochemical response after ursodeoxycholic acid treatment in patients with primary biliary cholangitis based on pretreatment nonenhanced MRI. Eur. Radiology 34 (2), 1268–1279. 10.1007/s00330-023-10080-w 37581659 PMC10853298

[B33] Zhou S. Li J. Liu J. Dong S. Chen N. Ran Y. (2024). Depressive symptom as a risk factor for cirrhosis in patients with primary biliary cholangitis: analysis based on Lasso-logistic regression and decision tree models. Behavior 14 (8), e3639. 10.1002/brb3.3639 39099389 PMC11298689

[B34] Zhu H. Zheng M. He H. Lei H. Tai W. Yang J. (2024). Development and external validation of an early prediction model to identify non-responsive patients and prognosis of UDCA treatment in primary biliary cholangitis. Sci. Rep. 14 (1), 31369. 10.1038/s41598-024-82854-1 39732944 PMC11682153

