# Supplementary material for: Development and evaluation of explainable machine learning models for predicting prognosis in patients with primary biliary cholangitis
Source: Front Mol Biosci. 2026 Jul 28;13:1900864. doi: 10.3389/fmolb.2026.1900864 (PMC13457136; doi:10.3389/fmolb.2026.1900864)
Supplement: Supplementary file 1 [file DataSheet1.pdf]

linear fitting coefficients (slope and intercept) of calibration curves:

Model	Training set		test set		External validation set	
	Slope	Intercept	Slope	Intercept	Slope	Intercept
Logistic Regression	0.9808	0.0137	0.9941	0.0042	0.9776	0.0269
Random Forest	1.2599	−0.0750	1.0848	−0.0400	1.1951	−0.0515
ExtraTrees	1.3709	−0.1213	1.1286	−0.0547	1.2952	−0.0929
XGBoost	1.0964	−0.0278	0.9752	0.0047	1.0747	−0.0134
LightGBM	1.2582	−0.0899	1.0528	−0.0360	1.1936	−0.0615
MLP	0.8255	−0.0754	0.8247	−0.0708	0.8075	−0.0450

confusion_matrix of all models:

========== ExtraTrees ==========

【Training Set】

Training Set confusion_matrix：

TN:3590 FP:78

FN:276 TP:1589

```
[[3590   78]
 [ 276 1589]]
```

Accuracy: 0.9360 (95%CI: 0.9292-0.9423)

Sensitivity: 0.8520 (95%CI: 0.8353-0.8692)

Precision: 0.9532 (95%CI: 0.9427-0.9630)

Specificity: 0.9787 (95%CI: 0.9740-0.9833)

F1_Score: 0.8998 (95%CI: 0.8893-0.9105)

【Test Set】

Test Set confusion_matrix：

TN:1442 FP:130

FN:246 TP:554

```
[[1442  130]
 [ 246  554]]
```

Accuracy: 0.8415 (95%CI: 0.8267-0.8558)

Sensitivity: 0.6925 (95%CI: 0.6622-0.7255)

Precision: 0.8099 (95%CI: 0.7799-0.8387)

Specificity: 0.9173 (95%CI: 0.9032-0.9304)

F1_Score: 0.7466 (95%CI: 0.7220-0.7716)

【External Validation Set】

External Validation Set confusion_matrix：

TN:1472 FP:58

FN:163 TP:679

```
[[1472   58]
```

[163   679]]
Accuracy: 0.9068 (95%CI: 0.8950-0.9182)
Sensitivity: 0.8064 (95%CI: 0.7803-0.8335)
Precision: 0.9213 (95%CI: 0.9008-0.9399)
Specificity: 0.9621 (95%CI: 0.9524-0.9710)
F1_Score: 0.8600 (95%CI: 0.8406-0.8781)

Training XGBoost...

========== XGBoost ==========
【Training Set】

Training Set confusion_matrix：
TN:3443   FP:225
FN:490   TP:1375
[[3443   225]
 [490 1375]]
Accuracy: 0.8708 (95%CI: 0.8612-0.8787)
Sensitivity: 0.7373 (95%CI: 0.7168-0.7568)
Precision: 0.8594 (95%CI: 0.8429-0.8758)
Specificity: 0.9387 (95%CI: 0.9310-0.9457)
F1_Score: 0.7937 (95%CI: 0.7785-0.8074)
【Test Set】

Test Set confusion_matrix：
TN:1426   FP:146
FN:234   TP:566
[[1426   146]
 [234   566]]
Accuracy: 0.8398 (95%CI: 0.8255-0.8546)
Sensitivity: 0.7075 (95%CI: 0.6771-0.7399)
Precision: 0.7949 (95%CI: 0.7649-0.8230)
Specificity: 0.9071 (95%CI: 0.8927-0.9203)
F1_Score: 0.7487 (95%CI: 0.7250-0.7725)
【External Validation Set】

External Validation Set confusion_matrix：
TN:1421   FP:109
FN:211   TP:631
[[1421   109]
 [211   631]]
Accuracy: 0.8651 (95%CI: 0.8516-0.8794)
Sensitivity: 0.7494 (95%CI: 0.7197-0.7797)
Precision: 0.8527 (95%CI: 0.8276-0.8794)

Specificity: 0.9288 (95%CI: 0.9154-0.9415)
F1_Score: 0.7977 (95%CI: 0.7746-0.8200)

Training LightGBM...

========== LightGBM ==========
【Training Set】

Training Set confusion_matrix：
TN:3500 FP:168
FN:392 TP:1473
[[3500 168]
[392 1473]]
Accuracy: 0.8988 (95%CI: 0.8903-0.9064)
Sensitivity: 0.7898 (95%CI: 0.7723-0.8090)
Precision: 0.8976 (95%CI: 0.8831-0.9121)
Specificity: 0.9542 (95%CI: 0.9473-0.9610)
F1_Score: 0.8403 (95%CI: 0.8274-0.8534)
【Test Set】

Test Set confusion_matrix：
TN:1421 FP:151
FN:240 TP:560
[[1421 151]
[240 560]]
Accuracy: 0.8352 (95%CI: 0.8204-0.8503)
Sensitivity: 0.7000 (95%CI: 0.6700-0.7312)
Precision: 0.7876 (95%CI: 0.7571-0.8201)
Specificity: 0.9039 (95%CI: 0.8891-0.9182)
F1_Score: 0.7412 (95%CI: 0.7172-0.7662)
【External Validation Set】

External Validation Set confusion_matrix：
TN:1435 FP:95
FN:191 TP:651
[[1435 95]
[191 651]]
Accuracy: 0.8794 (95%CI: 0.8672-0.8933)
Sensitivity: 0.7732 (95%CI: 0.7439-0.8030)
Precision: 0.8727 (95%CI: 0.8475-0.8973)
Specificity: 0.9379 (95%CI: 0.9251-0.9499)
F1_Score: 0.8199 (95%CI: 0.7987-0.8412)

Training Logistic Regression...

========== Logistic Regression ==========
【Training Set】

Training Set confusion_matrix：
TN:3346   FP:322
FN:854   TP:1011
[[3346   322]
 [854 1011]]
Accuracy: 0.7875 (95%CI: 0.7770-0.7979)
Sensitivity: 0.5421 (95%CI: 0.5198-0.5662)
Precision: 0.7584 (95%CI: 0.7356-0.7810)
Specificity: 0.9122 (95%CI: 0.9032-0.9207)
F1_Score: 0.6323 (95%CI: 0.6119-0.6531)
【Test Set】

Test Set confusion_matrix：
TN:1428   FP:144
FN:347   TP:453
[[1428   144]
 [347   453]]
Accuracy: 0.7930 (95%CI: 0.7774-0.8086)
Sensitivity: 0.5663 (95%CI: 0.5339-0.6008)
Precision: 0.7588 (95%CI: 0.7251-0.7939)
Specificity: 0.9084 (95%CI: 0.8938-0.9230)
F1_Score: 0.6485 (95%CI: 0.6208-0.6761)
【External Validation Set】

External Validation Set confusion_matrix：
TN:1399   FP:131
FN:372   TP:470
[[1399   131]
 [372   470]]
Accuracy: 0.7879 (95%CI: 0.7728-0.8052)
Sensitivity: 0.5582 (95%CI: 0.5237-0.5914)
Precision: 0.7820 (95%CI: 0.7496-0.8168)
Specificity: 0.9144 (95%CI: 0.9014-0.9284)
F1_Score: 0.6514 (95%CI: 0.6209-0.6812)

Training Random Forest...

========== Random Forest ==========
【Training Set】

Training Set confusion_matrix：
TN:3552　FP:116
FN:416　TP:1449
[[3552　116]
 [416 1449]]
Accuracy: 0.9038 (95%CI: 0.8955-0.9111)
Sensitivity: 0.7769 (95%CI: 0.7578-0.7971)
Precision: 0.9259 (95%CI: 0.9132-0.9380)
Specificity: 0.9684 (95%CI: 0.9627-0.9739)
F1_Score: 0.8449 (95%CI: 0.8322-0.8580)
【Test Set】

Test Set confusion_matrix：
TN:1450　FP:122
FN:252　TP:548
[[1450　122]
 [252　548]]
Accuracy: 0.8423 (95%CI: 0.8267-0.8567)
Sensitivity: 0.6850 (95%CI: 0.6526-0.7184)
Precision: 0.8179 (95%CI: 0.7889-0.8464)
Specificity: 0.9224 (95%CI: 0.9096-0.9345)
F1_Score: 0.7456 (95%CI: 0.7208-0.7701)
【External Validation Set】

External Validation Set confusion_matrix：
TN:1457　FP:73
FN:207　TP:635
[[1457　 73]
 [207　635]]
Accuracy: 0.8820 (95%CI: 0.8689-0.8955)
Sensitivity: 0.7542 (95%CI: 0.7248-0.7840)
Precision: 0.8969 (95%CI: 0.8750-0.9198)
Specificity: 0.9523 (95%CI: 0.9413-0.9625)
F1_Score: 0.8194 (95%CI: 0.7968-0.8416)

Training MLP...

========== MLP ==========
【Training Set】

Training Set confusion_matrix：
TN:2792　FP:876
FN:459　TP:1406
[[2792　876]

[459 1406]]
Accuracy: 0.7587 (95%CI: 0.7475-0.7697)
Sensitivity: 0.7539 (95%CI: 0.7332-0.7740)
Precision: 0.6161 (95%CI: 0.5959-0.6367)
Specificity: 0.7612 (95%CI: 0.7469-0.7748)
F1_Score: 0.6781 (95%CI: 0.6608-0.6950)
【Test Set】

Test Set confusion_matrix：
TN:1183   FP:389
FN:191   TP:609
[[1183   389]
[191   609]]
Accuracy: 0.7555 (95%CI: 0.7386-0.7728)
Sensitivity: 0.7612 (95%CI: 0.7332-0.7915)
Precision: 0.6102 (95%CI: 0.5808-0.6408)
Specificity: 0.7525 (95%CI: 0.7318-0.7742)
F1_Score: 0.6774 (95%CI: 0.6521-0.7010)
【External Validation Set】

External Validation Set confusion_matrix：
TN:1164   FP:366
FN:196   TP:646
[[1164   366]
[196   646]]
Accuracy: 0.7631 (95%CI: 0.7462-0.7808)
Sensitivity: 0.7672 (95%CI: 0.7399-0.7952)
Precision: 0.6383 (95%CI: 0.6069-0.6680)
Specificity: 0.7608 (95%CI: 0.7409-0.7819)
F1_Score: 0.6969 (95%CI: 0.6703-0.7213)
